# Perinatal outcomes in Finnish twins: a retrospective study

**DOI:** 10.1186/s12884-019-2670-3

**Published:** 2019-12-31

**Authors:** Annu-Riikka Susanna Rissanen, Riina Maria Jernman, Mika Gissler, Irmeli Katriina Nupponen, Mika Erkki Nuutila

**Affiliations:** 1 Department of Obstetrics and Gynecology, University of Helsinki and Welfare District of Päijät-Häme, Keskussairaalankatu 7, 15850 Lahti, Finland; 2 Obstetrics and Gynecology, University of Helsinki and Helsinki University Hospital, Haartmaninkatu 2, PL 140, 00029 HUS Helsinki, Finland; 3 Finnish Institute for Health and Welfare, Mannerheimintie 166, PL 30, 00271 Helsinki, Finland; 4 Karolinska Institute, Stockholm, Sweden; 5 Children’s Hospital, University of Helsinki and Helsinki University Hospital, Stenbäckinkatu 9, PL 347, 00029 HUS Helsinki, Finland

**Keywords:** Twins, Perinatal mortality, Pregnancy, multiple pregnancy, high-risk, Neonatal mortality

## Abstract

**Background:**

To establish the changes in perinatal morbidity and mortality in twin pregnancies in Finland, a retrospective register research was conducted. Our extensive data from a 28-year study period provide important information on the outcome of twin pregnancies in Finland that has previously not been reported to this extent.

**Methods:**

All 23,498 twin pregnancies with 46,996 children born in Finland during 1987–2014 were included in the study. Data were gathered from the Medical Birth Register and the Hospital Discharge Register (Finnish Institute for Health and Welfare, Finland) regarding perinatal mortality (PNM) and morbidity. For statistical analysis, binomial regression analysis and crosstabs were performed. The results are expressed in means, percentages and ranges with comparison to singletons when appropriate. Odds ratios from binomial regression analysis are reported. A *p*-value <0.05 was considered statistically significant.

**Results:**

There were 46,363 liveborn and 633 stillborn twins in Finland during 1987–2014. Perinatal mortality decreased markedly, from 45.1 to 6.5 per 1000 for twin A and from 54.1 to 11.9 per 1000 for twin B during the study period. Yet, the PNM difference between twin A and B remained. Early neonatal mortality did not differ between twins, but has decreased in both. Asphyxia, respiratory distress syndrome, need for antibiotics and Neonatal Intensive Care Unit (NICU) stay were markedly more common in twin B.

**Conclusions:**

In Finland, PNM and early neonatal mortality in twins decreased significantly during 1987–2014 and are nowadays very low. However, twin B still faces more complications. The outline provided may be used to further improve the monitoring and thus perinatal outcome of twins, especially twin B.

## Background

The number of twin pregnancies peaked in the late 1990s due to enhanced use of artificial reproductive technology (ART). Double joy? Hardly, as roughly all pregnancy complications are more common when carrying twins, and special attention is needed during pregnancy and childbirth [1]. Factors underlying the need for fertility treatment, such as advanced maternal age and increasing obesity, further add to the risks [2–5]. Finland pioneered in adopting a single-embryo policy and cryopreservation and thus, the proportion of twin pregnancies in Finland has reduced to approximately 1.4% [6, 7]. As the population is ethnically homogenous, the registers considered reliable and all twin pregnancies and deliveries managed in public hospitals, Finland is ideal for register-based research. Yet, there exist neither any published data on the perinatal outcome nor national guidelines for the management of twin pregnancies.

The overall PNM rate is higher for twins compared to singletons. This is mostly due to the fact that approximately half of twins are born preterm. When comparing preterm singletons and twins, the PNM difference is not as clear [8]. Prematurity and low birthweight cause morbidity and result in longer hospital stay, requiring also long-term follow-up [9]. Monochorionic twin pregnancies include specific risks related to a shared placenta, more than doubling the PNM rate compared to dichorionic twins [10].

Despite the remarkable evolvement in the management of premature and low birthweight newborns, the second twin has been reported to face adverse perinatal outcomes independent of chorionicity, sex and presentation [11]. The aim of this study was to establish the perinatal outcomes of twin pregnancies in Finland, separating data on twin A and B and comparing to singletons when possible.

## Methods

This is a retrospective study focusing on the perinatal outcomes of twin pregnancies in Finland in the years 1987–2014. The data were collected from the national Medical Birth Register and the Care Register (Finnish Institute for Health and Welfare, Finland), separating data on twin pregnancies. The Medical Birth Register contains, from 1987 onwards, data on all births in Finland, including information on deliveries and newborns until the age of seven days. Data on ART use and induction of labour are available since 1990, and data on neonatal diagnoses from 1996 onwards. The Hospital Discharge Register, also called the Care Register for Health Care, includes data on diagnoses, procedures and interventions received by the patients. Information on hospital outpatient care is available since 1998. Linkages between the registers can be performed by using the mother’s and child’s unique personal identity codes. Using International Statistical Classification of Diseases and Related Health Problems (ICD-9 in 1987–1995, ICD-10 since 1996) and Nordic Medico-Statistical Committee (NOMESCO) Classification on Surgical Procedures (NCSP) since 1997, the following data were retrieved and analyzed: the incidence of twin pregnancies (spontaneous and by ART), preterm deliveries (< 37 weeks of gestation), the onset of labour (spontaneous and induced), gestational age, sex, birthweight, Apgar scores at 1 and 5 min, umbilical artery pH, PNM, early neonatal mortality and neonatal morbidity. Intrauterine growth restriction and small-for-date are reported by the same ICD-code and thus, could not be separated in the registers. The mode of delivery is analyzed in further detail in our study on maternal complications in twin pregnancies [12].

Perinatal mortality rate was defined as death in the perinatal period (from 22 weeks of gestation up to 6 days postpartum) per 1000 children born dead or alive, and early neonatal mortality rate as death up to 6 days postpartum per 1000 children born alive. Stillbirths were defined from 22 weeks of gestation onwards. Neonatal morbidity includes diagnoses up to 28 days postpartum.

Data concerning the following perinatal/neonatal diagnoses were collected using corresponding ICD 10-codes: different levels of asphyxia (P20, P21.0, P21.1, P21.9), neonatal respiratory distress (P22), pneumothorax (P27.1), other respiratory distress (P28), retinopathy (H35.1) and sepsis caused by different pathogens (P36.0, P36.1, P36.2, P36.3, P36.8). Cerebral haemorrhages were collected by corresponding codes: (P52.0, P52.1, P52.2, P52.3, P52.4, P52.5, P52.6, P52.8) and data on necrotising colitis (P77), bowel perforation (P78.0), seizures (P90), hypoxis-ischemic encephalopathy grade I-III (P91.00, P91.01, P91.02), cerebral ischemia (P91.08), cystic periventricular and other leukomalasia (P91.1, P91.2), other and unspecified cerebral disturbance (P91.8, P91.9), were also collected. The proportions of low (< 2500 g) and very low (< 1500 g) birthweight and levels of prematurity were gathered from data on birthweight and gestational age at delivery.

In addition, the incidences of the following parameters were analyzed: neonatal antibiotic treatment, phototherapy, intubation, respiratory support, NICU stay, and children at home by the age of seven (7) days. All data were separated for twin A and B when possible and compared to corresponding singleton data from the same period, when applicable.

The data from the Medical Birth Register are available since 1987, except for ART, induction of labour and neonatal interventions since October 1990, and neonatal diagnoses since 1996. Five-minute Apgar scores were not recorded in the registers in 1990–2003, thus lacking during that period. Chorionicity was not recorded in the registers during the study period and thus could not be analyzed. Although the Finnish registers are considered reliable, the quality of reporting may vary causing possible bias. Twin parturients’ characteristics, and pregnancy and delivery complications have been presented in detail elsewhere [12].

### Statistical analyses

The data were analyzed using SPSS (IBM SPSS Statistics for Windows, Version 24.0, Armonk, NY, IBM Corporation) and RStudio (RStudio: Integrated Development Environment for R 1.1.447, Inc., Boston, MA 2016). Binomial regression for aggregate data was used to study predictors and dependencies of different variables. To further test the predictive properties of different models created, chi-squared test was used. A *p*-value <0.05 was considered statistically significant, and a 95% confidence interval was used. One sample t-test was used to compare the means of the variables and two-sample z-test was used to compare sample proportions. To analyze differencies between groups, crosstabs were also executed. Microsoft Excel 2010 and SPSS 24.0 were used to create figures, graphs and trend lines. The results are expressed in percentages, and the means, ranges and standard deviations are reported when appropriate.

## Results

During the years 1987–2014 there were a total of 23,498 twin deliveries (mean 839, range 645–958 per year) resulting in 46,363 livebirths and 633 stillbirths, altogether 46,996 newborns. Twin pregnancies accounted for 1.4% of all births in Finland (range 1.1–1.7%), peaking simultaneously with the rise of ART [12]. There were a total of 1,639,015 singleton deliveries (mean 58,537, range 53,779–64,923 per year) during the same period including 5805 singleton stillbirths and 1,633,210 livebirths.

During the study period, there were 23,254 livebirths for twin A (annual mean 831, range 631–950), of which 284 died at the age of < 7 days. For twin B, there were 23,109 livebirths (annual mean 825, range 621–948), of which 308 died at the age of < 7 days.

**Fig. 1:**
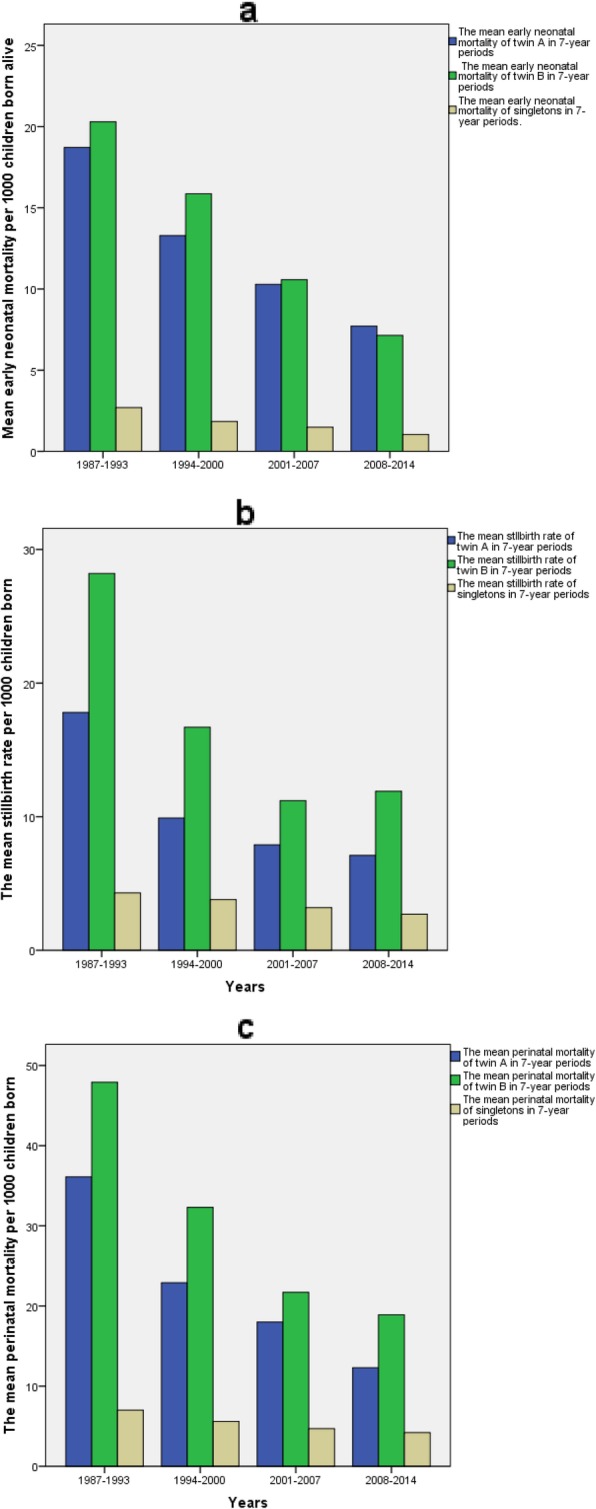
**a** Early neonatal mortality in twins per 1000 children born alive in Finland in 1987–2014 presented in 7-year periods. **b** Stillbirth rate of twins and singletons per 1000 children born in Finland in 1987–2014 presented in 7-year periods. **c** Perinatal mortality of twins and singletons born in Finland in 1987–2014 presented in 7-year periods

Early neonatal mortality did not differ between A and B, but has decreased markedly during the study period in both twins (from 22.4 to 2.6 per 1000 for twin A and from 26.5 to 3.9 per 1000 for twin B, *p* < 0.001). (Fig. 1a).

Twin A was stillborn in 244 and twin B in 389 cases. The stillbirth rate diminished in both twins (from 23.2 to 2.1 per 1000 for twin A and from 37.2 to 4.6 per 1000 for twin B until 2006 and remaining under 13.0 per 1000 since 2010, *p* < 0.001 for both), but remained significantly higher for twin B (p < 0.001) (Fig. 1b). Most of the stillbirths occurred during pregnancy with similar rates between the twins and compared to singletons (77.5% for twin A, 82.8% for twin B and 77.7% for singletons). Stillbirth during labour is a rare event in Finland with small numbers from 0 to 4 yearly for twin A, 0–7 for twin B and 2–44 for singletons with diminishing numbers during the study period. In twin A, PNM decreased from 45.1 to 6.5 per 1000, being highest in 1991 and remaining under 20.0 per 1000 after 2009. In twin B, PNM decreased from 54.1 to 11.9 per 1000 during the study period. In both twins, PNM has decreased significantly (*p* < 0.001 for both), but the PNM difference between twins has also remained statistically significant (*p* < 0.001) (Fig. 1c).

Low birth weight (< 2500 g) was reported in twin A on average in 38.6% of newborns (annual range 34.2–42.7%) and in twin B in 47.6% (annual range 42.3–52.6%). This difference is statistically significant (*p* < 0.001). The numbers are similar among live-born twins (39.0% for twin A and 48.5% for twin B). Very low birth weight (< 1500 g) was reported in twin A on average in 7.0% (annual range 5.0–10.2%) and in twin B in 8.0% (annual range 5.9–10.8%) with a significant difference between the twins (p < 0.001) even though the proportions do not differ markedly. The shares of very low birth weight live-born twins were comparable (7.0% for twin A and 8.1% for twin B).

In regression models analyzing factors affecting the PNM of twins, being twin B was a marked risk, whereas advancing time (from 1987 to 2014) significantly reduced the PNM (*p* < 0.001 for both). Very low birth weight had a significant effect on PNM when NICU stay was added to the model (*p* = 0.006) but also when the effect of very low birth weight, weight 1500–2499 g, weight ≥ 2500 g, advancing time and twin status were analyzed (*p* = 0.014). Including weight categories as proportions produced similar results (p = 0.006 and *p* = 0.007 when compared to ≥2500 g and 1500-2499 g weight categories). Sex had no influence on PNM or on asphyxia. When only the effect of the mode of delivery on PNM was analyzed among twins, Caesarean section seemed minorly protective (OR 0.995, CI 0.991–1.000, *p* = 0.043), but after adjusting for time (1987–2014) and twin status (A vs. B), it lost its significance (Table 1).

**Table 1:** Factors affecting perinatal mortality, binomial regression analysis

Variable	Estimate	OR	OR CI	p-value
Model 1*				
Twin B vs twin A	0.312	1.366	1.054–1.774	0.019
Advancing time	−0.038	0.963	0.951–0.975	< 0.001
< 1500 g	0.008	1.008	1.002–1.015	0.019
1500-2499 g	−0.002	0.998	0.996–1.001	0.277
≥ 2500 g	−0.000	1.000	0.998–1.001	0.675
*Model 2*				
Twin B vs twin A	0.230	1.258	1.089–1.455	0.002
Advancing time	−0.033	0.967	0.956–0.979	< 0.001
< 1500 g	0.010	1.010	1.003–1.017	0.006
NICU stay	−0.002	0.998	0.997–1.000	0.052
*Model 3*				
Twin B vs twin A	0.307	1.359	1.177–1.571	< 0.001
Advancing time	−0.042	0.959	0.948–0.969	< 0.001
Delivery: Vaginal attempt	−0.001	0.999	0.997–1.002	0.546
Delivery: Cesarean section	−0.002	0.998	0.996–1.001	0.250
Delivery: Combination delivery	0.002	1.002	0.994–1.010	0.622

*Similar results were obtained when weight categories were analyzed as shares

**Fig. 2:**
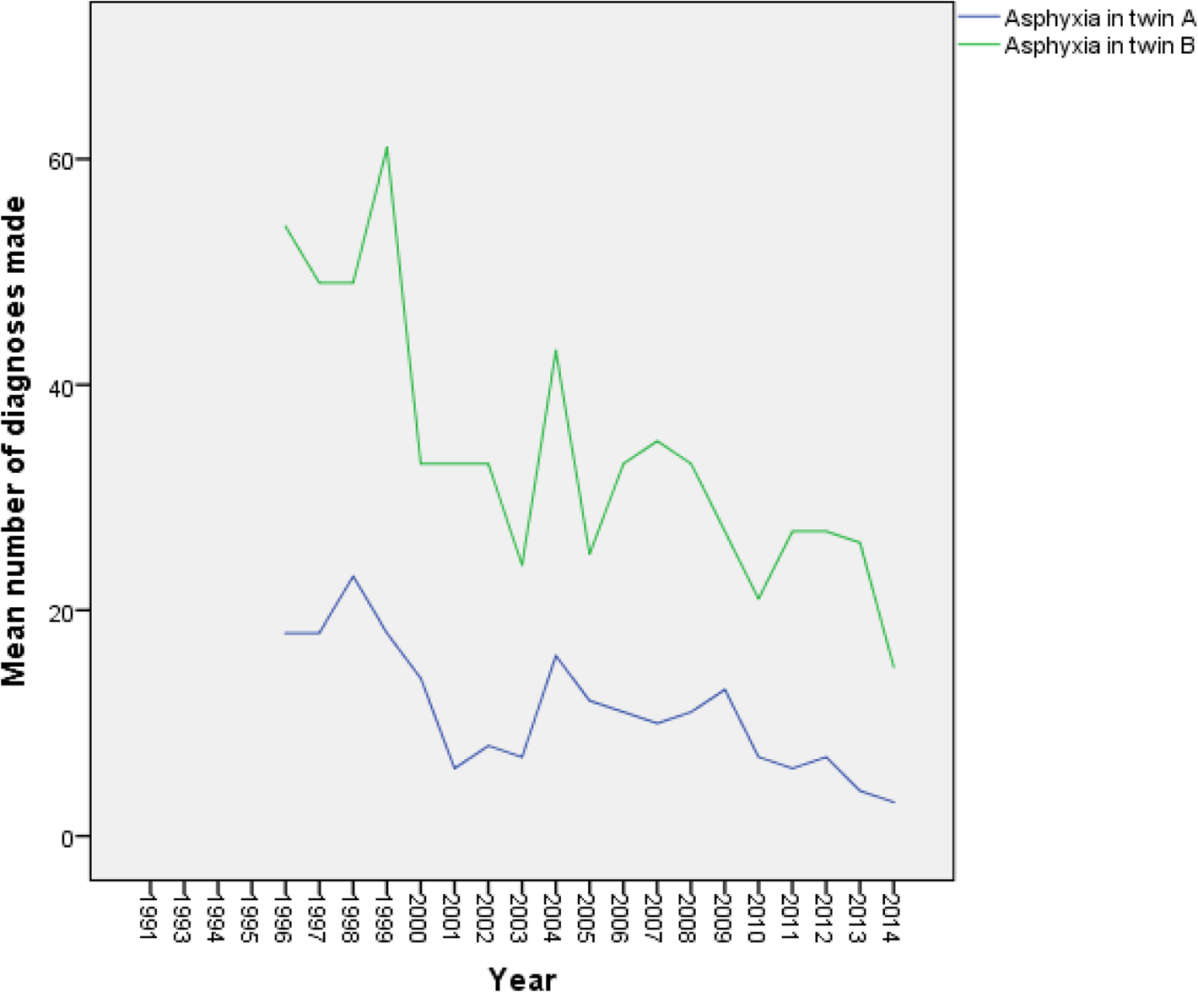
The mean number of twins diagnosed with any level of asphyxia in Finland in 1987–2014

Twins A and B did not differ significantly when neonatal enterocolitis diagnoses were analyzed, but the numbers were very small. Any level of asphyxia, was, however diagnosed markedly more often in twin B (*p* < 0.001) with marked reduction during the years among both twins (Fig. 2). The mode of delivery did not have any significant effect on the amount of asphyxias diagnosed among twins.

**Fig. 3:**
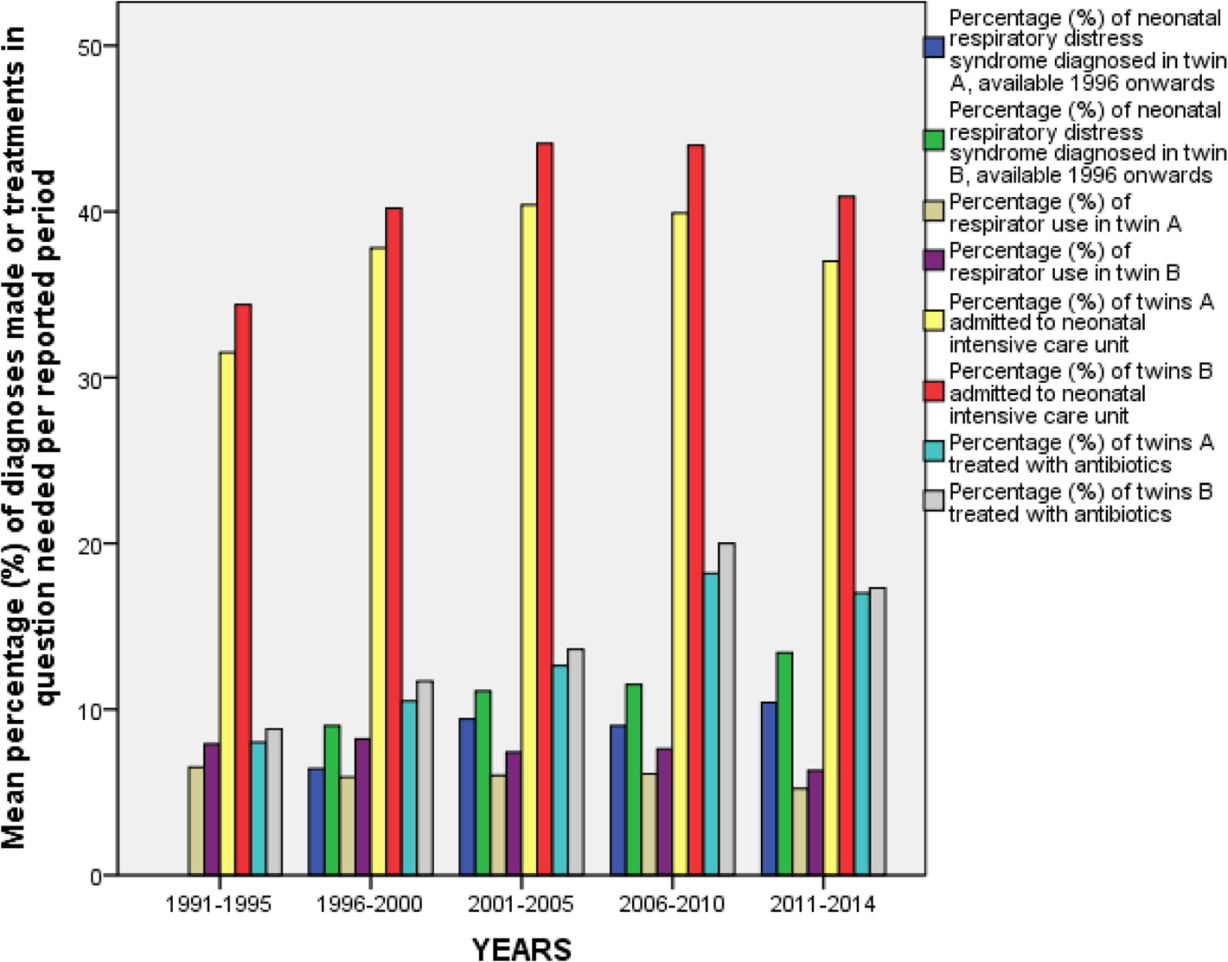
Neonatal diagnoses and treatments in Finland in 1991–2014 presented in 5-year and 4-year periods (2011–2014). The mean percentage of liveborn twins diagnosed with respiratory distress syndrome and twin newborns needing respirator, antibiotic use or admission to NICU

Respiratory distress syndrome was more common in twin B (*p* < 0.001). Twin B ended up in a respirator (p < 0.001) more often but the amount of bronchopulmonary dysplasia or hypoxic ischemic encephalopathy diagnoses did not differ between twin A and B. Twin B also received more antibiotics than twin A (*p* = 0.005) and was more often admitted to NICU (*p* < 0.001) but there was no significant change in the admittance rate to NICU during the years for neither of the twins (Fig. 3). Antibiotic use has markedly increased for both twins (p < 0.001) although sepsis diagnoses have remained stable. Phototherapy was needed equally for both twins and the amount of twin A vs. B at home by the age of seven days did not differ between twins.

During the study period, twin deliveries at 40–42 weeks of gestation decreased from 4.0 to 0.1% and deliveries at 37–39 weeks from 55.5 to 50.6%. Deliveries at 34–36 weeks increased from 25.7 to 33.3%. Deliveries before 34 weeks showed no significant change. Delivery was preterm in 44.9% (range 40.5–49.5%). Twin deliveries were induced in 27.7% of cases (range 20.4–38.0%). This proportion increased during the study period (*p* < 0.001). The mode of delivery had no significant effect on PNM in our material.

## Discussion

The purpose of this study was to create an outline of the trends and outcomes of twin pregnancies in Finland, focusing on perinatal morbidity and mortality. In general, PNM is very low in Finland and has showed a marked decrease during the study period in both twins, and among all newborns including singletons. However, PNM differences between twins A and B and between twins compared to singletons still remain. Coming last, twin B is more prone to complications during childbirth, but also during pregnancy as the stillbirth rate is also higher [13, 14]. Early neonatal mortality has, however, decreased for both twins during the study period with no significant difference between the twins. This not only highlights the difficulties in the decision-making of optimal timing of delivery in cases of fetal distress, but also the rapid evolvement of neonatal care [1, 15, 16]. Considering the amount of our data, these notions between the twins likely represent true differences rather than labelling bias. The higher risk for intrauterine complications and neonatal morbidity seem to be genuine problems for twin B despite strict monitoring during pregnancy and childbirth. The similarity in early neonatal mortality rate in both twins reflects the excellence of neonatal care in Finland and may also indicate that the gap between the twins could be narrowed or eventually even closed with timely antenatal interventions. As the time and cause of intrauterine demise is not available to us, the notion of higher intrauterine complications of twin B must, however, be evaluated with caution. Also, in rare cases, the demised twin may be mislabelled as twin B for convenience reasons. The information cannot be retrieved from the national registers. Although comparison of PNM rates between European countries is difficult due to differences in definitions and registration practices, fetal mortality rates vary considerably even when singletons are included. Nonetheless, fetal mortality in Finland seems to be among the lowest in Europe [17]. Although declining trends of PNM among twins were noticed also by Hehir et al., their reported mutual PNM rate for both twins remains higher than our results for either of the twins in the end of our study period [18]. Prematurity was the leading cause of PNM in their large cohort. Simultaneously, they noted an increase in the rate of Caesarean section. In our material, however, the mode of delivery did not affect PNM, and the CS rate in twins in Finland is lower (increase from 42 to 47% during 1987–2014, presented elsewhere) than the reported 62% in the study by Hehir et al. [12, 18].

Generally, the higher PNM in twins compared to singletons is partly explained by twins being more often born preterm and for parents, the burden of having two babies with possible neurological impairment is considerable [18, 19]. Some problems related to prematurity may not arise until school-age, with continuing anxiety during long-term follow-up [9]. It is essential to provide these families with accurate facts and support. There are, however, reports on lower PNM in the preterm period among twins compared to singletons in corresponding gestational age [8]. This may be explained by the more intense follow-up of twin pregnancies and optimizing the timing and place of delivery with adequate preparation for preterm birth. Underlying causes for prematurity also differ between twins and singletons with resultant differences in perinatal morbidity and mortality [20–22]. The possible role of advanced maternal age on worse perinatal outcome of twins is difficult to separate from other contributing risk factors in twin pregnancies. It should be considered as an additional risk for perinatal morbidity though, as the share of twin parturients aged 35 years or more is rising [12].

In our material, very low birth weight was a risk factor for higher PNM when all birthweight categories were included, but problems related to prematurity and NICU stay should be considered as possible confounding factors. Moreover, the confounding effect of gestational age could not be analyzed as we did not have information on weight categories by gestational age. If the newborn is of very low birthweight and the gestational age is around the critical weeks of 23, active vs. comfort care is chosen in accordance with the parents’ wishes. Therefore, some very immature newborns will not enter the NICU and this might slightly affect the PNM rate. Most cases, however, are admitted there and the decision to withdraw treatment in desperate situations will arise later in the upcoming days or weeks.

Male newborns have been reported to have a higher risk for adverse perinatal outcomes, but in our study, no differences in asphyxia diagnoses or PNM were discovered [11, 23]. Caesarean section does not appear protective for asphyxia after adjusting for birth year, which is an important notion when counselling mothers for the mode of delivery. Trial of labour is considered safe for twins also in the literature and decisions on the mode of delivery should be based on long-term outcomes of twins and mothers, which still requires further studies [24–26].

In our material, the second twin had an increased risk for asphyxia, respiratory distress syndrome, NICU stay, and antibiotic and respirator need with resultant higher PNM. The general rise in antibiotic use is not explained by the number of septic diagnoses or NICU stay, reflecting possible profylactic or early use of antibiotics. Being home by the age of seven days did not differ between twins, but the short-term morbidity in particular seems to be higher for twin B with possible consequences on early interaction. This finding also emphasizes the outstanding neonatal care in Finland, although in rare cases potential mislabeling of twins may slightly alter our results. In monochorionic twins, the higher PNM and morbidity are particularly due to twin-to-twin transfusion syndrome [27]. We were unable to analyze the differences between chorionicities because this information is still not collected into the Finnish registers. This clearly is a limitation of the study, which the authors are unable to correct, but it will be addressed in our upcoming clinical reports. Armson et al., however, concluded that the worse perinatal outcome of the second twin is independent of chorionicity, presentation, infant sex, birthweight and prolonged delivery interval. Their report is consistent with ours also regarding higher numbers of asphyxia and respiratory distress syndrome irrespective of gestational age in the second twin [11].

The strength of our study is the extensive material from reliable registers and a long study period. Details on perinatal outcome in twins in Finland have not been previously reported to this extent.

## Conclusions

Advancements in antenatal and neonatal care have resulted in very low and declining PNM and early neonatal mortality rates in Finnish twins. Perinatal morbidity and mortality remain higher for twin B, highlighting the need for better surveillance and decision making on optimal timing of delivery of twins. Despite the remarkable evolvement in the management of premature and low birthweight newborns, more information is needed on the spesific pitfalls of twin pregnancies. Further, to minimize the morbidity and mortality gap between twins A and B, we need standardized protocols for antenatal and neonatal care, possibly with the help of national guidelines. More studies with detailed data including cause and time of death and chorionicity are thus needed. Our extensive material from a 28-year study period with comparison to singletons could be of aid for future researchers. Our data are applicable to other industrialized countries.

## Data Availability

The complete set of tables used during the current study is available from the corresponding author on reasonable request. The Finnish register data have been given for this specific study, and the data cannot be shared without authorization from the register keepers. More information on the authorization application to researchers who meet the criteria for access to confidential data can be found at https://thl.fi/fi/web/thlfi-en/statistics/information-for-researchers/authorisation-application (THL).

## Abbreviations

ART: Artificial reproductive technology
ICD: International statistical classification of diseases and related health problems
NICU: Neonatal Intensive Care Unit
PNM: Perinatal mortality
