# Correction to: Perinatal outcomes in Finnish twins: a retrospective study

**DOI:** 10.1186/s12884-020-03120-6

**Published:** 2020-07-30

**Authors:** Annu-Riikka Susanna Rissanen, Riina Maria Jernman, Mika Gissler, Irmeli Katriina Nupponen, Mika Erkki Nuutila

**Affiliations:** 1 Department of Obstetrics and Gynecology, University of Helsinki and Welfare District of Päijät-Häme, Keskussairaalankatu 7, 15850 Lahti, Finland; 2 Obstetrics and Gynecology, University of Helsinki and Helsinki University Hospital, Haartmaninkatu 2, PL 140, 00029 HUS Helsinki, Finland; 3 Finnish Institute for Health and Welfare, Mannerheimintie 166, PL 30, 00271 Helsinki, Finland; 4 Karolinska Institute, Stockholm, Sweden; 5 Children’s Hospital, University of Helsinki and Helsinki University Hospital, Stenbäckinkatu 9, PL347, 00029 HUS Helsinki, Finland

**Correction to: BMC Pregnancy Childbirth 2, 20 (2020)**

**https://doi.org/10.1186/s12884-019-2670-3**

Following publication of the original article [1], the authors identified some errors in the Results and Discussion sections.

The Results and Discussion sections have been replaced with the updated version and the changes have been highlighted in **bold typeface**.

**Results**

Paragraph 8

Respiratory distress syndrome was more common in twin B (*p* < 0.001). Twin B ended up in a respirator (*p* < 0.001) more often but the amount of bronchopulmonary dysplasia or hypoxic ischemic encephalopathy diagnoses did not differ between twin A and B. **Twin B also received more antibiotics than twin A (*p*** **= 0.002) and was more often admitted to NICU (*p*** **< 0.001) (Fig. 3) even though both the admittance rate to NICU and antibiotic use have risen markedly during the years for both twins (*p* < 0.001 for both). Sepsis diagnoses have, however, remained stable. Phototherapy was needed equally for both twins, but the amount of twin B at home by the age of seven days was significantly lower than twin A (*p*** **= 0.006).**

**Discussion**

Paragraph 5, sentence 3

**The** short-term morbidity in particular seems to be higher for twin B with possible consequences on early interaction.
